# Field and greenhouse evaluations of soil suppressiveness to *Heterodera glycines* in the Midwest corn-soybean production systems

**DOI:** 10.21307/jofnem-2019-032

**Published:** 2019-06-03

**Authors:** Weiming Hu, Eyob Kidane, Deborah A. Neher, Senyu Chen

**Affiliations:** 1 University of Minnesota Southern Research and Outreach Center, 35838, 120th Street, Waseca, MN, 56093; 2 Department of Plant and Soil Science, 63 Carrigan Drive, Burlington, VT, 05405

**Keywords:** Biological control, Captan, Crop rotation, Formaldehyde, *Heterodera glycines*, Nematode-suppressive soil, Soybean, Soybean cyst nematode, Streptomycin, Tillage

## Abstract

Soil suppressive to the soybean cyst nematode (SCN), a major yield-limiting pathogen of soybean, plays an important role in biological control. Field and greenhouse experiments were conducted to evaluate the effects of tillage, crop sequence, and biocide application on SCN suppression in corn-soybean cropping systems in Minnesota. The experiment was a split-plot design with no-tillage and conventional tillage as main plots, and six crop-biocide treatments (CRCS, CSCS, SSSS, SSSS + streptomycin, SSSS + captan, and SSSS + formaldehyde – the four letters represent crops in 2009 to 2012, respectively; C is corn, R is SCN-resistant soybean, and S is SCN-susceptible soybean) as subplots with four replicates. Soil samples were taken from each plot at planting, midseason, and harvest each year for SCN egg counts, and soybean yield was determined. In addition, soil samples collected from each plot at midseason were assayed for suppressiveness to SCN. Tillage had minimal effect on SCN population density and soybean yield. Annual rotation with corn reduced SCN population density, but also reduced soil suppressiveness as SCN egg population density increased in the following SCN-susceptible soybean compared with soybean monoculture. Rotation with SCN-resistant soybean and corn was the most effective in reducing SCN population density. The bactericide streptomycin did not affect SCN populations but the fungicide captan increased SCN population density. The biocide formaldehyde was the most effective in reducing the level of suppressiveness to SCN. The greenhouse study confirmed that the soil was suppressive to SCN, but failed to detect effects of tillage, crop sequence, and biocide field treatments. This study demonstrated that the soil in the fields was suppressive to the SCN, and biological agents, especially fungal antagonists, were involved in nematode suppression.

Soybean cyst nematode (SCN), *Heterodera glycines* Ichinohe, is widely distributed throughout most soybean producing regions in the world (Riggs, 2004). This nematode has become a major yield-limiting factor in soybean production and causes an estimated annual yield loss of about $1 billion in the USA (Koenning and Wrather, 2010). Crop rotation, cultural practices, resistant cultivars, and nematicides are employed to reduce soybean yield suppression caused by SCN. Particularly, rotation of SCN-susceptible soybean with non-host and SCN-resistant cultivars is considered the best method to manage SCN (Niblack and Chen, 2004; Niblack, 2005).

The beneficial effects of rotation of corn and soybean have been studied extensively, and in general, rotation increases the yields of both corn and soybean crops (Crookston et al., 1991). However, mechanisms of crop rotation are not fully understood. Suppression of pests and pathogens, including plant-parasitic nematodes, is probably one of the beneficial effects of rotation on soybean and corn yields (Grabau and Chen, 2016a, 2016b). Benefits of rotation crops that are non-hosts to SCN are well known. An early study showed that crop rotation reduces SCN population density and improves the soybean yield in an SCN-infested field, and greater yields are obtained with longer rotation with non-hosts (Sasser and Uzzell, 1991). However, corn is less effective than leguminous non-hosts (Miller et al., 2006; Warnke et al., 2006), and a single year of corn rotation may be insufficient for SCN management (Chen et al., 2001b).

Since the late 1980s, conservation tillage is used increasingly in the USA to limit soil erosion, preserve soil moisture during drought, improve water quality, increase organic matter, and reduce fuel costs (Fawcett and Towery, 2003; Noel and Wax, 2003). However, beneficial effects of using conservation tillage in managing plant-parasitic nematodes are inconsistent (McSorley, 1998). Several studies focus on the effects of tillage on SCN in the USA. In the southern USA, no-till generally reduces SCN population densities (Tyler et al., 1983, 1987; Edwards et al., 1988; Lawrence et al., 1990; Hershman and Bachi, 1995; Koenning et al., 1995; Donald et al., 2009). Inconsistent effects of tillage on SCN are reported from the north central USA. Reduction of SCN population densities by no-tillage is reported from field experiments in western Kentucky (Hershman and Bachi, 1995) and Indiana (Westphal et al., 2009). These individual experiments agree with a survey of the north central USA (Workneh et al., 1999). However, there is no effect or minimum effect of tillage on SCN population in Minnesota, even though the soybean yields are greater in fields of conventional tillage practice (Chen et al., 2001c; Chen, 2007b). In contrast, greater SCN reproduction is reported in no-tillage soils as compared with conventional tillage in Illinois (Noel and Wax, 2003) and Minnesota (Noel and Wax, 2003; Grabau et al., 2017). These differences may be due to different cropping systems, soil types, environmental conditions, and their interaction with the nematode.

Generally, agricultural soil has low levels of buffering against plant diseases. However, there are some soils which greatly suppress a specific pathogen and, most importantly, the suppressiveness can be transferred by small portions of soil (Westphal, 2005). Specific suppressive soil has been favored by scientists because of its potential role in biological control. Usually, specific suppressive soil is first noticed when a pathogen declines with long-term monoculture of a susceptible crop (Schroth and Hancock, 1982; Westphal, 2005). Soils suppressive to SCN are reported in a number of locations in the USA and other regions in the world (Carris et al., 1989; Liu and Wu, 1993; Kim and Riggs, 1994; Chen et al., 1996; Sun and Liu, 2000; Chen, 2007a; Bao et al., 2011). Although dozens of nematode-suppressive soils have been discovered, the relation between crop sequence and soil suppressiveness has not been fully investigated, not to mention the interaction between microbes and cultural practices. Investigating soil suppressiveness under different cultural practices and biocide treatments in the field and validating the suppression in a greenhouse study is the first essential step to reveal the mechanism of soil suppression. Subsequently, validation in a research field with demonstrated specific suppressiveness to SCN serves as an ideal model to study mechanisms of suppressiveness (Bao et al., 2011; Hu et al., 2017). This study is the first to test effects of agricultural cultural practices and biocide on soil suppressiveness under both greenhouse and field conditions, which provides invaluable information for biological control.

Biocide treatments using fumigants, such as methyl bromide and formaldehyde, or fungicides, such as captafol, successfully reduce or eliminate the soil suppressiveness in fields (Williams, 1969; Kerry et al., 1980; Crump and Kerry, 1987). While captafol is a general fungicide, methyl bromide and formaldehyde are broad-spectrum biocides which can kill bacteria, fungi, and other organisms in the soil. The overall aim of this study was to distinguish the roles of tillage, crop sequence, and biocide in SCN soil suppression. Specific objectives were twofold: (i) quantify the effects of tillage, crop sequence, and biocide on SCN population density and soybean yield in the nematode-suppressive fields; and (ii) validate field treatment effects on soil suppressiveness at two field locations and in a controlled greenhouse environment. The justification for using the three biocide treatments was to target microbial communities, and determine if fungi, bacterial, or both were involved in the suppression of nematode populations. The treatment effects on microbial communities were studied with cultural methods as well as amplicon-based metagenomic analysis; and the data are presented in separate publications (Hu et al., 2017).

## Materials and Methods

### Field sites

This research was conducted at two field sites in Waseca County in Southern Minnesota for four years. Site 1 was located at the University of Minnesota Southern Research and Outreach Center (44°04′21″ N 93°31′21″ W) in Waseca, Minnesota, which had been planted to soybean continuously for 37 years and no-tillage had been practiced for the past 11 years before the experiment was established in 2009. Site 2 was in a commercial field (43°52′38″ N 93°43′14″ W) in Minnesota Lake, Minnesota, which had been in soybean monoculture for more than 20 years by 2005 when the field was planted to corn for four years prior to this experiment. The soil at Site 1 was a Nicollet clay loam (fine loamy, mixed, mesic Aquic Hapludoll), and the soil at Site 2 was Webster clay loam (fine loamy, mixed, mesic Endoaquoll). The soils in both fields were demonstrated to be suppressive to SCN (Chen, 2007a; Bao et al., 2011).

### Experiment design

**Figure A1:**
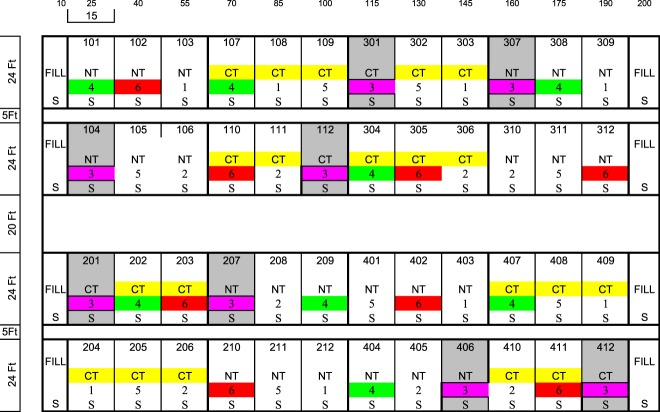
Field map of sample Site 1 in 2012.

The experiment was a split-plot design with no-tillage and conventional tillage as main plots, and the crop sequence-biocide treatments as subplots with four replicates (Fig. A1). The crop sequence-biocide treatments were carried out from 2009 to 2012 (Table A1): (i) corn (C)/soybean (S) (susceptible to SCN) annual rotation (C-S-C-S) without biocide, (ii) rotation of corn/SCN-resistant soybean (R)/corn/SCN-susceptible soybean (C-R-C-S) without biocide, (iii) monoculture of SCN-susceptible soybean (S-S-S-S) without biocide, (iv) S-S-S-S with bactericide streptomycin treatment, (v) S-S-S-S with captan (N-trichloromethylthio-4-cyclohexene-1, 2-dicarboximide) fungicide treatment, and (vi) S-S-S-S with the general biocide formaldehyde. At Site 2, the formaldehyde treatment was omitted considering its high toxicity and proximity of the site to a residential house. The main plot was 16.2 m long and 13.71 wide, each subplot was 7.6 m long and 4.57 m wide which included six rows of crops (Fig. A1). All treatments were repeated annually from 2009 to 2012 at both sites.

### Plot establishment and maintenance

For the formaldehyde treatment, 6.8 liter of 38% formaldehyde (Formalin) in 220 liters water was applied by irrigation in the four central rows (3000 L formalin per ha) 3 wk before planting. Appropriate safety instructions were followed when applying formalin. For the streptomycin and captan treatments, 18 grams of streptomycin sulfate (Sigma S 5601) (7.75 kg a.i./ha) and 27 grams a.i. of captan (80% wettable powder) (11.6 kg a.i./ha) each in 220 liters of water were applied by a pump from a tank into the surface of four central rows 1 wk before planting and every 2 wk after planting for two months (five times per year).

The conventional tillage treatment was fall chisel plowing after harvesting, and field cultivation followed by a finishing implement prior to planting. Fertilizer application was based on soil fertility test recommendations of the University of Minnesota Soil Test Laboratory. Fertilizer nitrogen in the form of urea with Agrotain nitrogen stabilizer was applied to corn plots only at the rate of 180 kg/ha in 2009, and 225 kg/ha in 2011. No other fertilizers were applied in soybean and corn during the 4yr. Corn and soybean were planted between late May and early June, and harvested between early October and mid-November depending on the soil and weather conditions each year. The SCN-resistant soybean cultivar was Latham EX547 RR N (PI 88788 source of resistance), SCN-susceptible soybean cultivar was Pioneer brand 92B13, and corn cultivar was DeKalb 46–61. All of the soybean and corn cultivars were resistant to glyphosate (2-phosphonomethylamino acetic acid), and glyphosate was used for both pre-emergence and post-emergence weed control. No insecticide or additional fungicide was used.

### Nematode population and soybean yield measurements

A soil sample consisting of 20 soil cores (2-cm diameter, 20 cm deep) was collected from each subplot in a systematic pattern across the two central rows at planting to assess initial (Pi), midseason (Pm) approximately 2 months after planting), and final population (Pf) at harvest. The soil was passed through a 5-mm aperture sieve and mixed thoroughly. The soil samples were stored in a cool room (4 °C) before being processed. Cysts were extracted from a subsample of 100 cm^3^ of soil with a semiautomatic elutriator (Byrd et al., 1976) and separated from soil particles and debris with centrifugation in a 63% (w/v) sucrose solution (Chen and Liu, 2005). Eggs were released from the cysts mechanically (Faghihi and Ferris, 2000) and collected in a 50-mL tube. The number of eggs was counted in a 0.5 to 2.0 mL aliquot, depending on the egg population density, and the total number of eggs in 100 cm^3^ of soil was derived. Soybean yields were measured from a 4.57-m length of the two central rows with a small plot combine. The soybean yield was standardized at 13% moisture.

### Greenhouse assay for nematode suppressiveness

To validate soil suppressiveness of each field treatment, soil samples from each plot were collected at midseason each year from 2009 to 2012 for a greenhouse assay. However, the results of 2011 were not reported because of insufficient SCN infection of soybean in the greenhouse. Approximately 4.5 kg soil was taken from 10 locations in each plot systematically to depth of 15 to 20 cm with a shovel. Each of the soil samples was passed through 5-mm aperture sieve, mixed thoroughly, and divided into three subsamples. Each subsample received one of three treatments: (i) 100% autoclaved field soil, (ii) 10% autoclaved field soil + 90% untreated field soil, and (iii) 100% field soil. The soil was autoclaved for 1 hr at 121°C.

An isolate of SCN HG Type 2.5.7 cultured on SCN-susceptible soybean in pots with autoclaved soil in the greenhouse was used as inoculum. The eggs of this population were extracted from the soil utilizing a similar method as described above, and then hatched in 4 mM ZnCl_2_ hatching solution (Chen et al., 2000a, 2000b). J2 that hatched within the second and fifth day were collected and rinsed thoroughly as inoculum.

Each subsample of soil was placed in a 15-cm-diameter pot. Seven soybean ‘Freeborn’ (PI 88788 source of resistance) seeds, that had been treated with 0.5% NaOCl for 3 min, were sown in each pot. Freeborn, which was susceptible to the population of HG 2.5.7 with a female index of 65% but resistant to the SCN populations (HG type 0) from the field plots, was used to minimize the effect of initial populations in the untreated soil. Pots were arranged in completely randomized blocks and maintained in the growth room with an average temperature of 28°C (range 20–30°C). After 1 wk, the plants were thinned to provide four plants, and 5,000 SCN J2 were added in six holes, 3 cm deep, around the soybean plants in each pot. The soil was supplied with a P-N-K fertilizer (0.08 g P_2_O_5_ + 0.0.08 g N + 0.04 g K_2_O/pot) after 4 wk of planting to minimize the effects of autoclaving on soil fertility.

Plant heights and total dry shoot weights per pot were measured at the termination of the experiments (60 d after inoculation). After cutting soybean shoots at the soil surface, the soil ball was broken and thoroughly mixed. Nematode egg population densities were determined with the procedures described previously (Chen and Liu, 2005).

### Data analysis

The general linear model (GLM) procedure in Statistical Analysis System (SAS) Version 9.2 (SAS Institute, Cary, NC) was used to perform the split-plot analysis of variance (ANOVA). Dependent variables were evaluated for normality and transformed as necessary before performing the ANOVA. Significant differences were reported at *P* < 0.05 unless otherwise stated. In the field experiments, egg population density was transformed by *x* ^0.2^ to *x* ^0.5^ and yield data were not transformed. In contrast, egg population density was transformed by log(*x*), and shoot dry weight data were not transformed in the greenhouse experiment.

## Results

### Nematode population density

#### Main effect of tillage

The overall mean SCN egg population density at planting in 2009 was 4,326 eggs/100 cm^3^ soil at Site 1 and only 102 eggs/100 cm^3^ soil at Site 2. No effect of tillage on the egg population density was observed in most of the 12 sampling occasions over the 4 yr at both sites, except that no-tillage reduced midseason egg population density at both sites in 2010, but increased midseason egg population density at Site 1 in 2012 as compared with conventional tillage (Table 1).

**Table 1:** Tillage, crop rotation, and biocide treatment effects on *Heterodera glycines* population density (eggs/100 cm^3^ soil) at nematode suppressive soil at two field sites in Minnesota.

Year		2009			2010			2011			2012		
Treatment		Pi	Pm	Pf	Pi	Pm	Pf	Pi	Pm	Pf	Pi	Pm	Pf
							Site 1						
Tillage:													
No-tillage		4,258 a	4,442 a	7,033 a	4,853 a	2,400 b	5,664 a	4,442 a	2,507 a	6,319 a	4,621 a	3,022 a	11,666 a
Conventional tillage		4,393 a	4,241 a	8,547 a	5,135 a	3,378 a	5,981 a	4,622 a	2,116 a	6,457 a	3,409 b	2,510 a	8,613 a
Crop-Biocide:													
C-S-C-S,	no biocide	3,881 a	3,200 c	2,250 b	1,766 b	1,102 d	4,238 c	2,263 c	1,275 b	1,175 d	677 d	1,114 c	11,803 b
C-R-C-S	no biocide	4,519 a	3,350 c	2,756 b	1,480 b	848 d	1,394 d	1,075 d	678 c	438 e	345 d	477 d	6,975 c
S-S-S-S	streptomycin	5,338 a	5,452 a	11,675 a	6,609 a	2,581 c	4,938 bc	3,556 b	1,791 b	6,488 c	4,413 bc	2,628 b	6,209 c
S-S-S-S	captan	4,013 a	5,409 a	10,444 a	6,775 a	4,131 b	6,381 b	4,334 b	2,406 b	9,181 b	6,206 ab	3,847 b	9,781 bc
S-S-S-S	no biocide	4,641 a	4,847 ab	9,794 a	5,947 a	2,828 c	5,331 bc	3,406 bc	1,597 b	6,506 c	3,969 c	2,666 b	8,169 c
S-S-S-S	formaldehyde	3,566 a	3,794 bc	9,825 a	7,391 a	5,856 a	12,656 a	12,563 a	6,125 a	14,541 a	8,481 a	5,866 a	17,897 a
Overall means		4,326	4,342	7,790	4,994	2,889	5,823	4,532	2,312	6,388	4,015	2,766	10,140
ANOVA (*F*-statistics):													
Tillage		0.09	0.01	3.82	0.19	25.46*	0.34	1.04	0.07	0.53	18.2*	0	1.14
Crop-Biocide		1.98	5.65***	23.08****	33.56****	32.2****	32.83****	38.2****	16.09****	75.89****	39.18****	33.03****	10.88****
Tillage × Crop-Biocide		0.32	1.84	2.38	1.28	1.67	3.76**	1.8	1.79	1.72	1.01	4.16**	1.23
							Site 2						
Tillage:													
No-tillage		139 a	553 a	1,146 a	899 a	519 b	2,586 a	2,892 a	2,747 a	6,708 a	3,635 a	2,368 a	7,646 a
Conventional tillage		65 a	539 a	1,732 a	803 a	955 a	2,893 a	2,702 a	2,467a	6,205 a	2,993 a	2,634 a	7,590 a
Crop-Biocide:													
C-S-C-S	no biocide	136 a	106 b	214 c	64 b	139 b	1,381 b	1,025 b	1,363 b	1,594 c	806 c	1,141 c	8,231 a
C-R-C-S	no biocide	123 a	158 b	91 c	374 b	72 b	153 c	163 c	100 c	125 d	144 d	197 d	3,631 b
S-S-S-S	streptomycin	92 a	1,053 a	1,586 b	802 a	1,064 a	4,506 a	3,919 a	4,322 a	10,369 ab	3,988 b	3,188 b	7,991 a
S-S-S-S	captan	98 a	809 a	2,430 ab	1,439 a	1,203 a	4,113 a	5,150 a	4,856 a	12,563 a	7,581 a	5,859 a	11,325 a
S-S-S-S	no biocide	61 a	602 a	2,873 a	1,579 a	1,208 a	3,544 a	2,781 a	3,347 a	7,631 b	4,050 b	2,119 b	6,913 a
Overall means		102	546	1,439	851	737	2,740	2,797	2,607	6,457	3,314	2,501	7,618
ANOVA (*F*-statistics):													
Tillage		3.9	0.41	2.07	1.64	10.56*	0.96	1.21	0.89	3.85	0.25	2.87	0.02
Biocide-crop			7.18***	22.01****	7.77***	22.45****	34.09****	20.98****	29.94****	54.87****	37.73****	28.89****	6.09**
Tillage × Crop-Biocide		0.09	0.26	1.08	0.15	4.67**	1.17	1.24	0.69	0.36	1.25	0.43	0.57

Note: C, corn; S, SCN-susceptible soybean, R, SCN-resistant soybean, and the letters in the order represent the crops from 2009 to 2010. Planting (Pi), midseason (Pm), and harvest (Pf); the data are main effect of the split-plot experiment with tillage as main plots and crop-biocide treatments as subplots with four replicates. The mean values followed by the contrasting letters in the column are significantly different according to the LSD test at *P* < 0.05. *P < 0.05; **P < 0.01; ***P < 0.001; ****P < 0.0001.

#### Main effect of crop sequence

The effect of soybean-corn annual rotation on SCN egg population density was observed over the 4 yr at both sites (Table 1). SCN egg population density fluctuated dynamically with annual corn-soybean rotation regardless of whether resistant or susceptible cultivars of soybean were growing. The SCN egg population density gradually decreased while corn was growing and increased when soybean was growing (Table 1). The soybean-corn annual rotation with resistant soybean always had lower egg populations than monoculture, but the egg population density rebounded in the fourth year in annual rotation with susceptible soybean growing (Table 1).

#### Main effect of biocide

The effect of biocide treatment on the SCN egg population density varied among biocides (Table 1). The bactericide streptomycin did not affect egg population density over the 4 yr at both sites, except reduced egg population density at harvest of 2009 at Site 2 (Table 1). Although varying among sampling seasons, the fungicide captan tended to increase SCN egg population density at both sites (Table 1). This effect started at the second year of the application at both sites and was significant in the later years (Table 1). For example, the increasing effect was significant at harvest of 2011, planting and midseason of 2012 at Site 2 (Table 1). At Site 1, formaldehyde consistently increased the egg population density from midseason of 2010 to harvest of 2012 (Table 1). Initially, the formalin treated plots had slightly lower egg populations than the monoculture control. However, by midseason of 2010, the egg population in formalin treated plots was at least 200% and sometimes 400% that of the control. By the end of the experiment in 2012, egg populations reached 17,897 eggs/100 cm^3^ soil, a level which can be considered conducive to SCN (Table 1).

### Interaction of tillage and crop sequence-biocide

There were no interactions between the tillage and crop-biocide treatments except for 3 of the 12 sampling times (Table 1). Significant interactions were observed at harvest 2010 and midseason 2012 at Site 1 and at midseason 2010 at Site 2 (Tables 1 and 2). There were sporadic differences between tillage for individual crop sequences and no consistency between sites or among years. For example, tillage increased SCN population density in streptomycin treated plots in fall 2010, and C-R-C-S with no biocide treatments in the midseason 2012 at Site 1. Tillage also increased egg population density in streptomycin treated plots in the midseason 2010 at Site 2. In contrast, conventional tillage decreased SCN population density in the S-S-S-S, captan treatment in the midseason 2012 at Site 1, and C-R-C-S, no biocide treatment in the midseason 2010 samples.

**Table 2:** Interactive effects of tillage and crop-biocide treatments on the population density (eggs/100 cm^3^ soil) of *Heterodera glycines*.

Treatments		Pf2010, Site 1				Pm2012, Site 1				Pm2010, Site 2			
Crop	Biocide	No-tillage		Conventional tillage		No-tillage		Conventional tillage		No-tillage		Conventional tillage	
C-S-C-S	No biocide	5,175	ab A	3,300	c A	959	c A	1,269	cd A	141	b A	138	c A
C-R-C-S	No biocide	1,238	d A	1,550	d A	219	d B	734	d A	131	b A	13	c B
S-S-S-S	Streptomycin	3,300	c B	6,575	b A	2,744	b A	2,513	b A	322	a B	1,806	bc A
S-S-S-S	Captan	5,538	b A	7,225	b A	5,438	a A	2,256	bc B	1,047	a A	1,359	a A
S-S-S-S	No biocide	4,275	bc A	6,388	b A	2,988	b A	2,344	bc A	956	a A	1,459	ab A
S-S-S-S	Formaldehyde	14,463	a A	10,850	a A	5,788	a A	5,944	a A				

Note: C, corn; S, SCN-susceptible soybean; R, SCN-resistant soybean, and the letters in the order represent the crops from 2009 to 2010. Pm = midseason, and Pf = harvest. The data are means of four replicates. The values followed by the contrasting lowercase letters in the same column or the values followed by the contrasting uppercase letters in the row within the same sampling time are different at *P* < 0.05 according to LSD test.

When data were analyzed for individual tillage treatments, the SCN population density was always greatest in the formaldehyde treatment at Site 1 (Table 2). Captan increased the SCN population density in the no-tillage plots at Site 1 only in the midseason of 2012 (Table 2). Streptomycin did not affect SCN egg population density at these three sampling occasions (Table 2). The C-R-C-S treatment reduced egg densities consistently. The SCN egg population density was progressively less in susceptible soybean grown in monoculture with conventional tillage, monoculture with no-tillage, and rotated with corn with conventional tillage (Table 2). In contrast, SCN egg population density was less in the no-till than conventional tillage in the C-S-C-S rotation at midseason 2012 at Site 1 (Table 2). The one year of corn in 2009 reduced SCN population density at the midseason 2010 susceptible soybean in both tillage treatments at Site 2 (Table 2).

### Soybean yield

**Figure 1:**
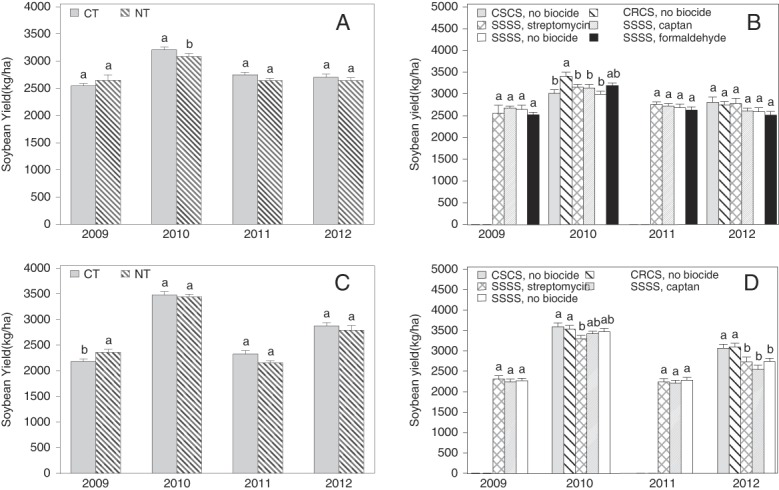
Tillage, crop rotation, and biocide treatment effects on soybean yield at two field sites infested with *Heterodera glycines* in Minnesota. (A) Tillage treatment at Site 1. (B) Biocide and crop sequence treatments at Site 1. (C) Tillage treatment at Site 2. (D) Biocide and crop sequence treatments at Site 2. CT = Conventional tillage, and NT = No-tillage. The CSCS, CRCS, and SSSS are 4-yr crop sequences where C is corn, S is SCN-susceptible soybean, R is SCN-resistant soybean, and the consecutive letters represent the crops in 2009, 2010, 2011, and 2012, respectively. Illustrated are mean (±1 SE) main effects (*n* = 4). Bars annotated by contrasting letter(s) within the same year are different according to LSD test at *P* ⩽ 0.05.

Conventional tillage increased soybean yield 3.89% in 2010 at Site 1 (Fig. 1A) and decreased soybean yield 7.38% in 2009 at Site 2 (Fig. 1C). No tillage effect on soybean yield was detected in any other year (Fig. 1A,C). At Site 1, biocide-crop sequence affected soybean yield only in 2010, resistant soybean plots had greater yield than susceptible soybean, but the plots with formaldehyde treatment had similar yield as resistant soybean (Fig. 1B). In the same year (2010), yield was less in monocultures of susceptible soybean treated with streptomycin than in susceptible soybean following corn or resistant soybean following corn (Fig. 1D). In 2012, the soybean yield was greater in plots with corn-soybean rotation than soybean monoculture, regardless of biocide treatments (Fig. 1D).

### Nematode suppressiveness in greenhouse assay

**Figure 2:**
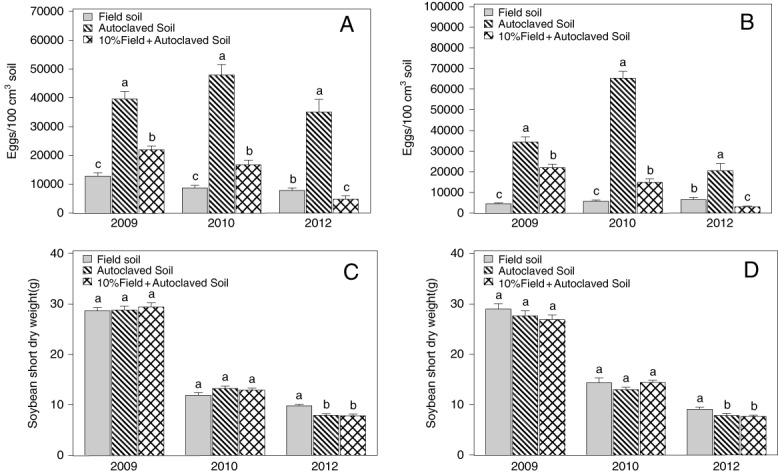
Egg population density of *Heterodera glycines* and soybean shoot dry weight in the greenhouse assay of soil from plots treated with tillage, crop rotation, and biocides at two field sites in Minnesota. (A) Egg population density at Site 1. (B) Egg population density at Site 2. (C) Soybean shoot dry weight at Site 1. (D) Soybean shoot dry weight at Site 2. The data are means of all tillage and crop-biocide treatments with four replicates (*n* = 48 at Site 1, and *n* = 40 at Site 2). The lines above the bars indicate the standard error. Bars annotated by contrasting letter(s) within the same year are significantly different according to LSD test at *P* ⩽ 0.05.

Soil from both sites treated with autoclave-heating dramatically reduced the level of nematode suppression as it increased SCN egg population densities in the soil two months after planting (Fig. 2A,B). The smallest egg population density was generally in the 100% untreated field soil. Adding 10% of the untreated field soil into autoclaved soil also dramatically reduced SCN egg population density as compared with the autoclaved soil. The treatment of 10% field soil mixed with 90% autoclaved soil had greater egg population densities in 2009 and 2010, but slightly reduced egg population densities in 2012, as compared with the 100% field soil (Fig. 2A,B). No crop rotation, tillage, or biocide effects on the egg population density were detected in the greenhouse assays. Neither was there a difference in plant dry weights in 2009 and 2010, but untreated field soil had greater dry weight than the other two treatments containing 10% or no untreated soil in 2012 (Fig. 2C,D).

## Discussion

In this study, we investigated the effect of tillage, crop rotation, and biocide treatments on SCN soil suppression in the field, and validated soil suppression in subsequent greenhouse bioassays. The effect of tillage on soybean yield was minimal and inconsistent. Conventional tillage increased soybean yield (3.89%) only in the second year at Site 1, but decreased soybean yield (7.38%), the first year at Site 2. This agrees with previous studies reporting an inconsistent, yet positive, effect of conventional tillage on soybean yield (Lueschen et al., 1992; Chen, 2007b). Tillage either had none or only a slight effect on SCN population density in both fields, indicating a minor effect of tillage on nematode soil suppression, which agree with previous studies of tillage effect on SCN population density in Minnesota (Chen et al., 2001c; Chen, 2007b; Grabau et al., 2017). Many factors such as time, environmental conditions, soil biotic and abiotic factors, cropping systems, crop residues, and SCN initial population density were presumed to be responsible for the effect of tillage on SCN population density. Previous studies suggested that tillage did not affect SCN population density in the first 4 yr (Chen et al., 2001c), and longer-term (5–10 yr) effect of tillage on SCN population density was also minimal (Chen, 2007b). A study of even longer (14–16 yr) tillage treatments suggests that SCN population densities are reduced by conventional tillage occasionally, but not affected by tillage at most sampling occasions (Grabau et al., 2017). One reason for inconsistent reports is that soil in no-tillage plots may become more compacted as time progresses and this process may take several years (Koenning et al., 1995).

Chen (2007a, 2007b) hypothesizes that different soil biological activities, including the activities of parasites and predators of nematodes, are at least partially responsible for the different tillage effects on SCN in northern and southern regions. The lower temperature and longer period of frozen soil in the northern states may result in less difference in the biological activities between no-tillage and conventional tillage. Parasitism of SCN J2, quantity of trapping fungi in the soil, and parasitism of SCN eggs in the two fields in the present study are similar in both conventional tillage and no-tillage (Hu et al. unpublished). Tillage affects a number of factors that can influence SCN and vary by site or time including soil moisture (Venterea et al., 2006), soil temperature (Licht and Al-Kaisi, 2005), soil structure (Licht and Al-Kaisi, 2005), and crop growth (Chen, 2007b). More research is needed to reveal the mechanisms behind the different tillage effects between the southern and northern regions.

Although the non-host corn reduced SCN population density, 1 yr of corn was not sufficient for SCN management, and when SCN-susceptible soybean was planted after the annual rotation of corn, SCN population density increased rapidly to the level similar to that in monoculture. This result was similar to a previous study (Chen et al., 2001b), which demonstrated that it may take about 5 yr of corn to reduce SCN population density to a non-damaging level in a field where the initial egg population density after harvesting soybean is 20,000 eggs/100 cm^3^ soil. This confirms that corn was relatively ineffective as a non-host in reducing SCN populations (Miller et al., 2006). Interestingly, the SCN population density at the end of experiment in the fall of 2012 at Site 1 in this study was greater in the annual rotation than the monoculture of SCN-susceptible soybean, probably because the annual rotation of corn reduced populations of antagonists of SCN and the level of soil suppressiveness. Rotation of corn and SCN-resistant soybean was the most effective crop sequence in reducing SCN population density in this study, agreeing with previous studies (Chen et al., 2001b).

Among the biocide treatments, the application of formaldehyde seemed to be the most effective for disrupting nematode suppressiveness. The increased SCN egg population density under formaldehyde treatment over the 4 yr supported the evidence of presence of antagonistic agents that attack nematodes in different stages – either egg, juvenile or adult – in the suppressive soil (Chen, 2007a). While captan increased SCN population density on a few sampling occasions at Site 1, there was no increase of SCN population density by streptomycin. The results suggest that fungi play a more important role than bacteria in SCN suppression. A previous greenhouse study also demonstrated the importance of fungal antagonists in suppression of SCN (Bao et al., 2011). However, an additional study of the microbial community in the soil collected from Site 1 suggests that both fungi and bacteria were involved in suppressing SCN population density (Hu et al., 2017). Several fungal and bacterial species are suggested to be important in SCN-suppressive soils in previous reports. For example, the sterile fungus ARF18 isolated from Arkansas has great suppressive potential on SCN eggs (Kim and Riggs, 1991). Bacterium *Pasteuria nishizawae* was responsible for an SCN-suppressive soil in Illinois (Noel et al., 2007). *Hirsutella rhossiliensis* and *H. minnesotensis* are frequently isolated from SCN J2 (Chen and Reese, 1999; Liu and Chen, 2000; Chen et al., 2000a, 2000b), and these two species of fungi are also frequently isolated from infective SCN J2 from the two fields in this study. However, the species and frequencies of the fungi isolated from J2 or eggs in this study were similar between biocide treatments (Hu et al., unpublished), and specific mechanisms involved in the suppression of SCN population are still not fully revealed.

There was no effect of biocide treatment effect on soybean yield. This indicates that the increase of SCN population density in the formaldehyde and captan treatments at Site 1 was not due to better growth of soybean and greater food source, and rather that the increase can be attributed to the reduction of biocontrol agents in the soil. Conversely, the increased SCN population density in the biocide treatments did not reduce the soybean yield at Site 1. This result was similar to previous studies in which there was no increase of soybean yield of resistant cultivars as compared with susceptible cultivars in the field (Chen et al., 2001a; Bao et al., 2013). The lack of significant response of soybean yield to SCN infestation in the sites was probably due to good soil fertility and biocontrol of SCN in the fields. However, the SCN-resistant cultivar in 2010 following corn yielded 13.0% better than the susceptible soybean either following corn or in monoculture at Site 1. The increased soybean yield might be due to both smaller SCN initial population density and the genetic resistance, but also could be due to difference in agronomic traits between the two soybean cultivars.

In the greenhouse assay of soil suppressiveness to nematodes, transferring a small amount of soil to conducive soil is one of the most commonly used methods to detect specific suppression in soil (Westphal, 2005; Chen, 2007a; Bent et al., 2008). This technique proved successful with 10% field soil in the greenhouse study. The results confirmed the previous studies that the soils were suppressive to the SCN (Chen, 2007a; Bao et al., 2011). However, we failed to detect any difference in level of SCN suppressiveness between the tillage treatments or among the crop-biocide treatments. This result suggests that these cultural practices and even the biocide treatments could not completely eliminate the organisms responsible for SCN suppression in the soil, and the method used in the greenhouse bioassay is not sensitive enough to detect the level of suppressiveness of soil containing similar groups of organisms. Adding 10% of field soil into the autoclaved conducive soil could allow any of the suppressive organisms to establish their population densities in the greenhouse even their population density in the field has been suppressed by a treatment such as crop rotation and formaldehyde application.

In conclusion, tillage had little effect on the soil suppressiveness. Annual rotation with corn reduced SCN population density, but also reduced the level of soil suppressiveness and increased SCN population density in the following SCN-susceptible soybean as compared with the soybean in monoculture. Rotation with SCN-resistant and non-host corn was the most effective in lowering SCN population density. The biocide formaldehyde was the most effective in reducing the level of suppressiveness and increasing SCN population density through time, while captan also increased SCN population density in some sampling occasions at Site 1. This field study demonstrated that the soil in the fields was suppressive to the SCN, and biological agents were involved in nematode suppression.

## Appendix

Table A1

**Table A1.** Plot treatments.

	Crop sequence				
Treatment	2009	2010	2011	2012	Biocide treatment
1	C	S	C	S	No
2	C	R1	C	S	No
3	S	S	S	S	Streptomycin, every year
4	S	S	S	S	Captan, every year
5	S	S	S	S	No
6	S	S	S	S	Formalin^a^

Note: ^a^Site 1 only; Crops: C = corn (2011, DK 46-61); R1 = SCN-resistant soybean ‘Latham EX547 RR N’ (PI 88788 SR); S = SCN-susceptible soybean ‘Pioneer brand 92B13’; All crops are roundup-ready.

Figure A1
